# Emergency Department Visits Among Patients With Dementia Before and After Diagnosis

**DOI:** 10.1001/jamanetworkopen.2024.39421

**Published:** 2024-10-14

**Authors:** Cameron J. Gettel, Yuxiao Song, Craig Rothenberg, Courtney Kitchen, Andrea Gilmore-Bykovskyi, Terri R. Fried, Abraham A. Brody, Stephanie Nothelle, Jennifer L. Wolff, Arjun K. Venkatesh

**Affiliations:** 1 Department of Emergency Medicine, Yale School of Medicine, New Haven, Connecticut; 2 Center for Outcomes Research and Evaluation, Yale School of Medicine, New Haven, Connecticut; 3 BerbeeWalsh Department of Emergency Medicine, University of Wisconsin–Madison School of Medicine & Public Health, Madison; 4 Section of Geriatrics, Department of Internal Medicine, Yale School of Medicine, New Haven, Connecticut; 5 VA Connecticut Healthcare System, West Haven; 6 Rory Meyers College of Nursing, New York University, New York, New York; 7 Division of Geriatric Medicine and Palliative Care, New York University Grossman School of Medicine, New York, New York; 8 Division of Geriatric Medicine and Gerontology, Department of Medicine, Johns Hopkins University School of Medicine, Baltimore, Maryland; 9 Department of Health Policy and Management, Johns Hopkins Bloomberg School of Public Health, Baltimore, Maryland; 10 Roger C. Lipitz Center for Integrated Health Care, Johns Hopkins Bloomberg School of Public Health, Baltimore, Maryland

## Abstract

This cohort study assesses changes in emergency department (ED) use among Medicare beneficiaries aged 65 years and older before and after receiving a diagnosis of dementia.

## Introduction

Emergency department (ED) visits among persons living with dementia represent a substantial health care challenge, often necessitating targeted interventions to optimize care and support.^1^ Previous studies have primarily focused on health care use outcomes after formal diagnosis or during end-of-life periods for individuals with dementia.^2,3^ We assessed changes in ED use before and after incident dementia diagnosis among Medicare beneficiaries aged 65 years and older.

## Methods

This cohort study used data from the Medicare Current Beneficiary Survey from 2015 to 2021 and included beneficiaries with an incident diagnosis of dementia (ie, cases) and a 1:2 propensity score–matched (for age, sex, race and ethnicity, and chronic conditions) population without a dementia diagnosis (ie, comparators). We identified cases from inpatient or outpatient claims with a diagnosis of dementia (*International Statistical Classification of Diseases, Tenth Revision* codes F01, F02, F03, and G30). The Yale University Institutional Review Board waived the need for approval and informed consent owing to the use of publicly available data. We followed the STROBE reporting guideline.

The primary outcome was the rate of monthly ED visits for cases and comparators in the 6 months before and 6 months after the date that case patients were diagnosed with dementia. If a dementia diagnosis was received on the same date as an ED visit, we classified these patients as having the visit before the diagnosis. We calculated descriptive statistics and estimated a zero-inflated negative binomial regression model, including adjustments for key beneficiary sociodemographic and health status characteristics. Self-reported race and ethnicity data were collected because these variables are associated with disparities in health care access and use.

Statistical analyses were conducted at the beneficiary level using R, version 4.0.2 (R Project for Statistical Computing) and were performed October 1, 2023, through June 30, 2024. Two-sided *t* tests or Pearson χ^2^ tests established statistical significance at *P* < .05.

## Results

Our sample included 1779 Medicare beneficiaries with incident dementia and 3558 matched comparators without dementia, reflecting 17 919 504 Medicare beneficiaries after applying survey weights. Among patients with an incident dementia diagnosis, the mean (SD) age at the time of diagnosis was 82.0 (7.6) years; 1060 patients (59.6%) were women and 719 (40.4%) were men. The patient characteristic percentages for patients with a dementia diagnosis were similar to those for patients without a diagnosis (Table).

**Table.** Patient Characteristics^a^

Characteristic	Patient group		*P* value^b^
	No dementia diagnosis (n = 3558)	Dementia diagnosis (n = 1779)	
Age, mean (SD), y	81.9 (7.6)	82.0 (7.6)	>.99
Race and ethnicity			
White	2966 (83.4)	1473 (82.8)	.60
Racial or ethnic minority^c^	592 (16.6)	306 (17.2)	
Sex			
Women	2119 (59.6)	1060 (59.6)	>.99
Men	1439 (40.4)	719 (40.4)	
No. of chronic conditions			
<2	1811 (50.9)	747 (42.00)	<.001
≥2	1747 (49.1)	1032 (58.0)	
Income level			
Low	1102 (31.0)	712 (40.0)	<.001
Middle	1047 (29.4)	444 (25.0)	
High	1409 (39.6)	623 (35.0)	
Marital status			
Single/separated/widowed	2121 (59.6)	1141 (64.1)	.001
Married	1437 (40.4)	638 (35.9)	
Education			
High school	794 (22.3)	494 (27.8)	<.001
Post high school	2764 (77.7)	1285 (72.2)	
IADL impairments			
<2	2621 (73.7)	1013 (56.9)	<.001
≥2	937 (26.3)	766 (43.1)	
ADL impairments			
<2	2683 (75.4)	1138 (64.0)	<.001
≥2	875 (24.6)	641 (36.0)	

Abbreviations: ADL, activities of daily living; IADL, instrumental activities of daily living; NA, not applicable.

^a^ Unless noted otherwise, data are presented as No. (%) of patients.

^b^ Calculated via *t* test or Pearson χ^2^ test.

^c^ Includes the Medicare Current Beneficiary Survey categories Asian, Black or African American, Hispanic, other, and unknown; this category was combined due to survey response volume and for consistency with previous Medicare Current Beneficiary Survey studies.

In the sixth month before a diagnosis of dementia, the ED visit rate was 1.69 out of 100 beneficiaries vs 2.08 out of 100 beneficiaries for those without a diagnosis. In the month immediately preceding a diagnosis of dementia, the ED visit rate was 13.0 out of 100 beneficiaries vs 2.95 out of 100 beneficiaries for those without a diagnosis. In the month immediately subsequent to a diagnosis of dementia, the ED visit rate was 3.32 out of 100 beneficiaries vs 2.73 out of 100 beneficiaries for those without a diagnosis (Figure, A). Within the entirety of the 12-month period assessed, having a diagnosis of dementia was associated with a 40% increase in the likelihood of having an ED visit (odds ratio, 1.40 [95% CI, 1.25-1.58]; *P* < .001) (Figure, B).

**Figure.**
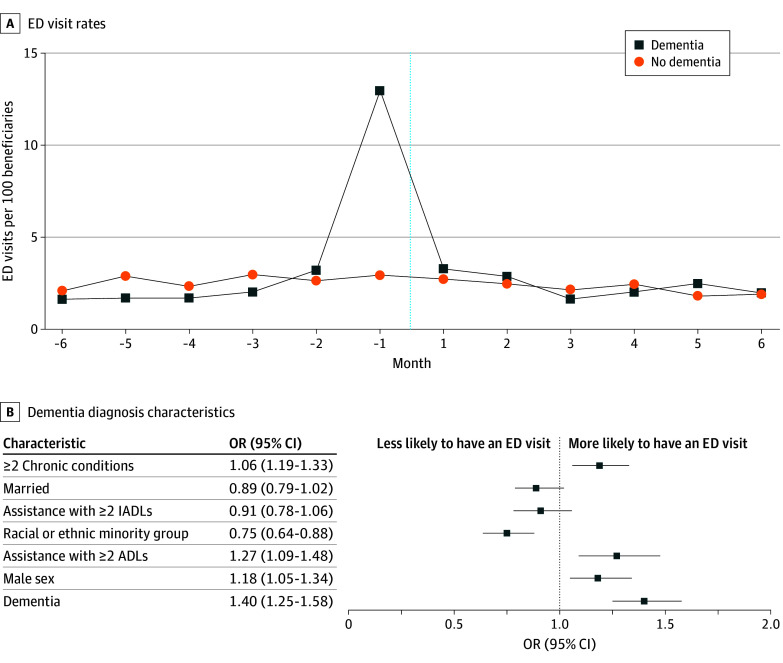
Emergency Department (ED) Visit Rates and Outcomes Among Patients With and Without a Dementia Diagnosis A, ED visit rates among patients with and without a dementia diagnosis. The dotted line delineates the date of diagnosis. B, Forest plot of patient characteristics and their associations with ED visit outcomes within the 12-month period of dementia diagnosis. ADL indicates activities of daily living; IADL, instrumental activities of daily living; and OR, odds ratio.

## Discussion

The observed changes in ED use, with peaks before and after dementia diagnosis, suggest that the diagnostic process may precipitate acute health care crises and increased health care–seeking behavior among individuals with dementia and their caregivers. Additionally, ED visits may trigger a diagnostic cascade toward dementia, reflecting the complex nature of dementia identification and management. Often a crucial contact point, the ED setting provides a valuable opportunity to screen for cognitive impairment,^4,5,6^ which might otherwise go unnoticed until it has progressed substantially.

A limitation of this study is that the generalizability of our findings may not extend beyond Medicare beneficiaries. Additionally, claims-based dementia diagnoses may not capture milder forms of cognitive impairment. Future longitudinal and qualitative studies are needed to elucidate the complex associations between dementia progression, comorbidities, and health care use.

## Notes

Supplement.
Data Sharing Statement
