# Supplementary material for: Emergency Department Visits Among Patients With Dementia Before and After Diagnosis
Source: JAMA Netw Open. Author manuscript; Available in PMC 2024 Dec 2. (PMC11581500; doi:10.1001/jamanetworkopen.2024.39421)
Supplement: Supplement [file NIHMS2035092-supplement-Supplement.pdf]

Data Sharing Statement

Gettel. Emergency Department Visits Among Patients With Dementia Before and After Diagnosis. *JAMA Netw Open*. Published October 14, 2024.
doi:10.1001/jamanetworkopen.2024.39421

Data
Data available: Yes
Data types: Deidentified participant data, Data dictionary
How to access data: cameron.gettel@yale.edu
When available: With publication

Supporting Documents
Document types: Statistical/analytic code
How to access documents: cameron.gettel@yale.edu
When available: With publication

Additional Information
Who can access the data: Researchers whose proposed use of the data has been approved.
Types of analyses: For specified purposes determined by the author team.
Mechanisms of data availability: After approval of a proposal.
